# SARS-CoV-2 seroprevalence in pregnant women in Kilifi, Kenya from March 2020 to March 2022

**DOI:** 10.3389/fpubh.2023.1292932

**Published:** 2023-12-19

**Authors:** Angela Koech, Geoffrey Omuse, Alex G. Mugo, Isaac G. Mwaniki, Joseph M. Mutunga, Moses W. Mukhanya, Onesmus Wanje, Grace M. Mwashigadi, Geoffrey G. Katana, Rachel Craik, Peter von Dadelszen, Kirsty Le Doare, Marleen Temmerman, periCOVID-Africa, Bridget Freyne, Kondwani Kawaza, Samantha Lissauer, Halvor Sommerfelt, Melanie Etti, Philippa Musoke, Robert Mboizi, Stephen Cose, Victoria Nankabirwa, Lauren Hookham, Joseph Ouma, Gordon Rukondo, Madeleine Cochet, Merryn Voysey, Liberty Cantrell, The PRECISE Network, Patricia Okiro, Consolata Juma, Marvin Ochieng, Emily Mwadime, Esperança Sevene, Corssino Tchavana, Salesio Macuacua, Anifa Vala, Helena Boene, Lazaro Quimice, Sonia Maculuve, Eusebio Macete, Inacio Mandomando, Carla Carillho, Umberto D’Alessandro, Anna Roca, Hawanatu Jah, Andrew Prentice, Melisa Martinez-Alvarez, Brahima Diallo, Abdul Sesay, Sambou Suso, Baboucarr Njie, Fatima Touray, Yahaya Idris, Fatoumata Kongira, Modou F.S. Ndure, Gibril Gabbidon, Lawrence Gibba, Abdoulie Bah, Yorro Bah, Laura A. Magee, Hiten Mistry, Marie-Laure Volvert, Thomas Mendy, Lucilla Poston, Jane Sandall, Rachel Tribe, Sophie Moore, Tatiana T. Salisbury, Donna Russell, Prestige T. Makanga, Liberty Makacha, Reason Mlambo, Aris Papageorghiou, Alison Noble, Hannah Blencowe, Veronique Filippi, Joy Lawn, Matt Silver, Joseph Waiswa, Ursula Gazeley, Judith Cartwright, Guy Whitley, Sanjeev Krishna, Marianne Vidler, Jing (Larry) Li, Jeff Bone, Mai-Lei (Maggie) W Kinshella, Domena Tu, Ash Sandhu, Kelly Pickerill, Ben Barratt

**Affiliations:** ^1^Centre of Excellence in Women and Child Health, Aga Khan University, Nairobi, Kenya; ^2^Department of Obstetrics and Gynaecology, Aga Khan University, Nairobi, Kenya; ^3^Department of Pathology, Aga Khan University, Nairobi, Kenya; ^4^Kilifi County Department of Health and Sanitation Services, Kilifi, Kenya; ^5^Department of Women and Children’s Health, Kings College London, London, United Kingdom; ^6^Faculty of Medicine and Health Sciences, Ghent University, Ghent, Belgium; ^7^St. George’s University of London, London, United Kingdom

**Keywords:** SARS-CoV-2, COVID-19, seroprevalence, pregnancy, Kenya, antibodies

## Abstract

**Background:**

Seroprevalence studies are an alternative approach to estimating the extent of transmission of SARS-CoV-2 and the evolution of the pandemic in different geographical settings. We aimed to determine the SARS-CoV-2 seroprevalence from March 2020 to March 2022 in a rural and urban setting in Kilifi County, Kenya.

**Methods:**

We obtained representative random samples of stored serum from a pregnancy cohort study for the period March 2020 to March 2022 and tested for antibodies against the spike protein using a qualitative SARS-CoV-2 ELISA kit (Wantai, total antibodies). All positive samples were retested for anti-SARS-CoV-2 anti-nucleocapsid antibodies (Euroimmun, ELISA kits, NCP, qualitative, IgG) and anti-spike protein antibodies (Euroimmun, ELISA kits, QuantiVac; quantitative, IgG).

**Results:**

A total of 2,495 (of 4,703 available) samples were tested. There was an overall trend of increasing seropositivity from a low of 0% [95% CI 0–0.06] in March 2020 to a high of 89.4% [95% CI 83.36–93.82] in Feb 2022. Of the Wantai test-positive samples, 59.7% (95% CI 57.06–62.34) tested positive by the Euroimmun anti-SARS-CoV-2 NCP test and 75.9% (95% CI 73.55–78.17) tested positive by the Euroimmun anti-SARS-CoV-2 QuantiVac test. No differences were observed between the urban and rural hospital but villages adjacent to the major highway traversing the study area had a higher seroprevalence.

**Conclusion:**

Anti-SARS-CoV-2 seroprevalence rose rapidly, with most of the population exposed to SARS-CoV-2 within 23 months of the first cases. The high cumulative seroprevalence suggests greater population exposure to SARS-CoV-2 than that reported from surveillance data.

## 1 Introduction

The SARS-CoV-2 pandemic has shown significant variability in its evolution in different geographical settings (1). Understanding the regional evolution of the pandemic is important to provide insights into the spread and ultimate burden and impact of disease. This can inform governments’ investments in mitigation measures as they plan for future health shocks.

The primary approach to describing the epidemiology of SARS-CoV-2 infections relied on surveillance systems that conducted active case finding, testing and contact tracing (2). This approach misses many cases as most SARS-CoV-2 infections are asymptomatic (3). Limited laboratory capacity for confirmatory SARS-CoV-2 tests, evolving testing policies, and increasing population fatigue with testing limited both the usefulness and accuracy of this approach (3). An alternative approach to monitoring population trends is to conduct population-based serosurveillance. The geographical pattern of spread can be described, and a better estimate of cumulative incidence obtained (4). This is particularly useful in remote settings where access to confirmatory polymerase chain reaction (PCR) testing is poor and where it is harder for public health surveillance teams to reach people (3, 4).

Serosurveillance typically relies on household level surveys. However, these studies are costly and may have been hampered by early pandemic movement restrictions. Conducting serological surveys on proxy samples of the population may enable inferences to be made on the population at lower costs, provided that the sampled population is representative of the general population. In general, pregnant women are socially and economically active and their interactions with other members of the community are important for studying infectious diseases like SARS-CoV-2. Compared to sampling other persons seeking care from health facilities, sampling of pregnant women is preferred as they more often attend these facilities for routine pregnancy care and delivery and not due to illness. Such studies are even more representative if antenatal care attendance and facility delivery rates of the population are high. Routine blood sampling in antenatal care offers an opportunity for serological testing without requiring additional blood draws (5). Various studies in Africa and beyond have sampled pregnant women presenting to health facilities for antenatal care and/or delivery for both SARS-CoV-2 (6–9) and other infections (10).

In this study, we used stored serum samples collected in a prospective pregnancy cohort study to determine SARS-CoV-2 seroprevalence. The pregnancy cohort study was initiated before the pandemic and allowed evaluation of the changes in seropositivity over time from the early phases of the pandemic, creating repeated cross-sectional observations in the same geographic area to establish trends in an evolving pandemic (11). The findings can be correlated with secondary data on the pandemic (e.g., public health mitigation measures, emergence of SARS-CoV-2 variants and data on confirmed cases of COVID-19) to identify factors that modified changes in seroprevalence.

The study objectives were to: (1) determine the trend in SARS-CoV-2 seroprevalence in pregnant women in Kilifi County from March 2020 to March 2022; (2) assess any associations between the seroprevalence and geographic characteristics in the study area; and (3) correlate the seroprevalence with data regarding confirmed SARS-CoV-2 cases in Kenya and with public health mitigation measures at the various time points.

## 2 Methods

### 2.1 Study design and setting

**Figure 1:**
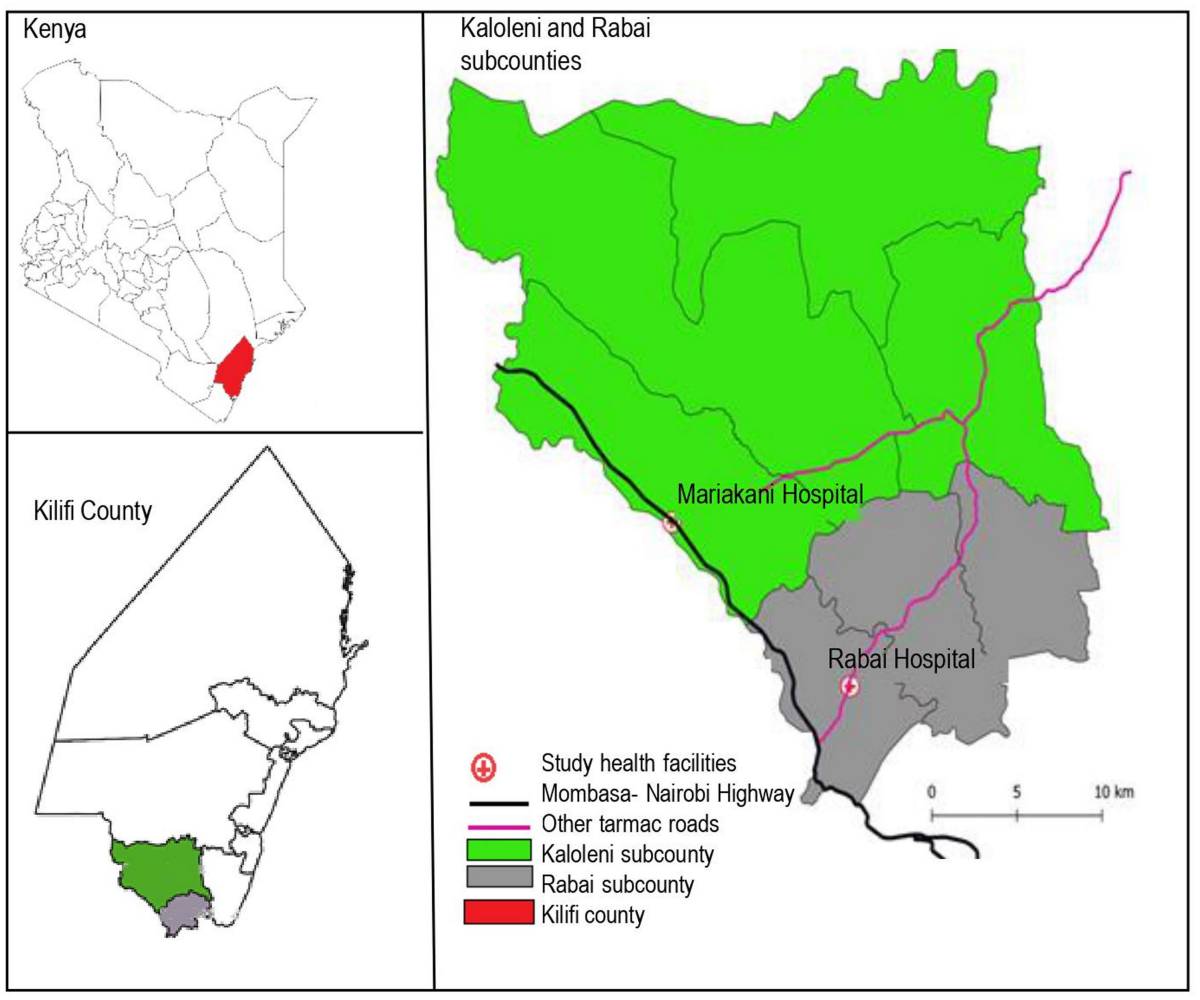
Map of Kaloleni and Rabai Sub-counties.

This seroprevalence study was nested within a prospective observational multi-country cohort study, the PREgnancy Care Integrating translational Science Everywhere (PRECISE) Network study. The PRECISE network has established a repository of biological samples with matching clinical and non-clinical data to evaluate placental disorders (hypertension, fetal growth restriction, preterm birth and stillbirth) in sub-Saharan Africa (12, 13). In Kenya, enrolment took place at the rural Rabai Hospital (formerly Rabai Health Centre) and the urban Mariakani Hospital in Kilifi County, coastal Kenya. Both hospitals are level 3 facilities offering primary care to local communities while serving as referral centers for other facilities in Rabai and Kaloleni Sub-counties, respectively. Both are public facilities managed by the Kilifi County Department of Health and Sanitation Services that offer antenatal care and comprehensive emergency obstetric and neonatal care (14). Kaloleni and Rabai Sub-counties have a combined area of 892.3km^2^ and a population of over 240,000 people (15). Antenatal care attendance in Kilifi is high with 99.3% achieving at least 1 visit (16). A major highway (The Mombasa-Nairobi highway, A109) traverses the study area and abuts the Mariakani Hospital (Figure 1).

The PRECISE study enrolment period was from 24th June 2019 to 6th December 2022 with a pause in study activity from 16th March 2020 to 23rd August 2020 due to the COVID-19 pandemic. A health care worker strike led to a reduction in enrolment in January 2021.

Pregnant women aged 16 to 49 years who presented for routine antenatal care or who presented to health facilities with pregnancy complications (hypertension, suspected fetal growth restriction and intrauterine fetal death/stillbirth) were enrolled (13). All participants gave written informed consent for study participation, biological sample collection and storage and use of the data and samples for future research. Clinical and non-clinical data were collected during each visit. This included the address and village of residence.

### 2.2 The nested seroprevalence study

The nested seroprevalence analysis reported here drew samples from March 2020 to March 2022. The final dataset used for this study was accessed on 30th December 2022.

We aimed to obtain representative samples to enable estimation of a monthly seroprevalence over the defined study period. For samples collected in the antenatal period, we selected at random 40% of samples for months with more than 100 collections and 100% of samples for months with 100 or fewer collections. For samples collected at delivery, we selected all available samples. A participant could be selected more than once in different months. However, participants with more than one sample within the same month (duplicates) were identified and only the most recent sample used for analysis.

### 2.3 Sample collection, pre-processing, and storage

Maternal venous blood was collected at antenatal clinic visits and within 48 h of delivery. Serum aliquots were stored in plain FluidX tubes (Azenta Life Sciences, Massachusetts, USA) and immediately stored in −80°C freezers at the study sites. The samples were shipped periodically in dry ice to the main biorepository at Aga Khan University Hospital Nairobi for long term storage in −80°C freezers.

### 2.4 Laboratory analysis

Laboratory testing for SARS-CoV-2 antibodies was undertaken at the Aga Khan University Hospital using the ETI-MAX 3000 (Diasorin, Saluggia, Italy), a fully automated micro titer plate analyzer. We tested for SARS-CoV-2 antibodies against the spike protein using a qualitative Wantai SARS-CoV-2 enzyme linked immunosorbent assay (ELISA) kit (Beijing Wantai Biological Pharmacy Enterprise Co., Ltd., Beijing, China) that detects total antibody concentrations. The manufacturer reported assay sensitivity is 94.36%, with a specificity of 100%. All specimens that tested positive using this assay were tested using anti-SARS-CoV-2 NCP ELISA and Anti-SARS-CoV-2 QuantiVac ELISA (Euroimmun, Lübeck, Germany) kits for the detection of IgG antibodies to SARS-CoV-2 nucleocapsid (NCP) and spike (S1 and S2) proteins, respectively. Euroimmun Anti-SARS-CoV-2 NCP ELISA (IgG) is a semi-quantitative immunoassay with a reported sensitivity of 94.6% and specificity of 99.8% in samples collected at-least 10 days after confirmed SARS-CoV-2 infection. The anti-SARS-CoV-2 QuantiVac ELISA (IgG) is a quantitative immunoassay for the determination of the concentration of IgG antibodies against the S1 antigen including the receptor binding domain of SARS-CoV-2 in serum in a broad linear range using a 6-point calibration curve. The assay’s sensitivity after >21 days past symptoms is 93.2% and specificity of 99.8%.

### 2.5 Quality control

The three SARS-CoV-2 ELISA assays were determined to be 100% sensitive and 100% specific using 20 known negative samples collected prior to the COVID-19 pandemic and 22 positive samples (confirmed PCR positive clinical cases with spike and nucleocapsid antibodies). In addition, we conducted a SARS-CoV-2 serologic assessment using randomly selected serum aliquots from the PRECISE cohort from the pre-pandemic period (July to December 2019, maximum 20 samples per month, total 113) using the Wantai anti-SARS-CoV-2 ELISA to assess its specificity. These samples were processed and stored with the same procedures. All 113 samples were negative for SARS-CoV-2 total antibody.

### 2.6 Data analysis

We used Stata 16.1 (StataCorp LLC, College Station, TX, United States) for statistical analyzes. Seroprevalence was determined for each calendar month as a proportion [% 95% confidence interval, (CI)] of samples testing positive over all samples tested in that calendar month. Scatter graphs were used to demonstrate temporal trends. No statistical tests for trend were undertaken.

We determined *a priori* not to make comparisons based on individual characteristics but rather on geographic characteristics: the health facility of enrolment and the location of village of residence relative to the Mombasa-Nairobi highway. Any villages directly traversed or touched by the highway were considered to be ‘along the highway.’ Odds ratios were obtained, and comparisons made using χ^2^ test. We adjusted for the effect of time defined as each month in the study period. Statistical significance was set at *p* < 0.05.

### 2.7 Comparisons between the monthly seroprevalence, COVID-19 cases in Kenya and public health measures

We obtained publicly available data on SARS-CoV-2 specific public health interventions from press releases on the national government Ministry of Health website (17). We determined *a priori* to focus solely on interventions likely to have impact on the spread of infection. Specifically, we were interested in restriction of travel at both national and county level, limiting attendance to learning institutions and rolling out of SARS-CoV-2 vaccination. We consulted Kilifi County Department of Health and Sanitation Services officials to obtain data on county-specific public health interventions. We obtained publicly available data (18) on the daily number of cases in Kenya in the study period (17) and made graphical comparisons between these data, the monthly PRECISE seroprevalence data, and selected public health interventions.

## 3 Results

**Figure 2:**
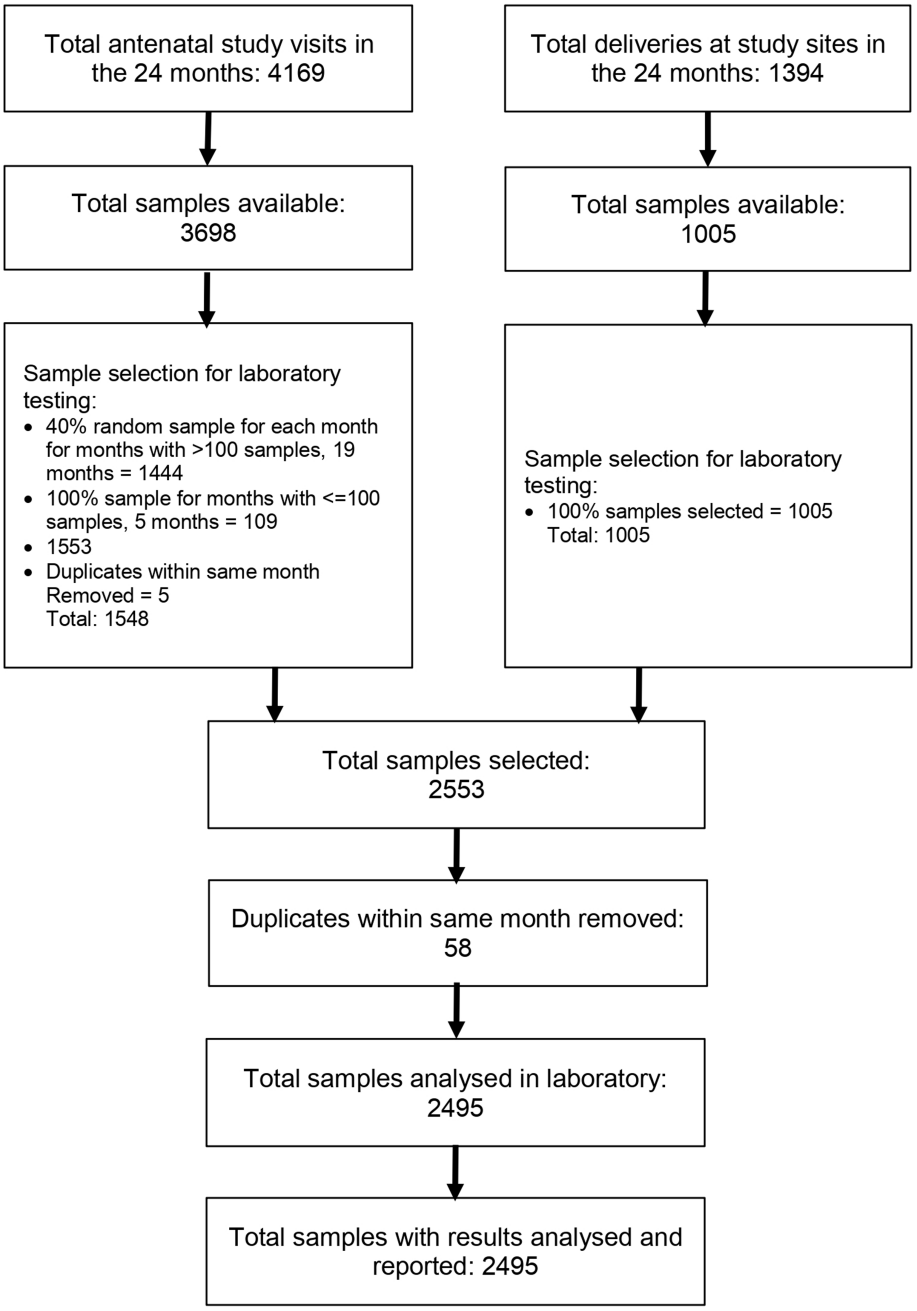
Study flow diagram showing sample selection for analysis.

During the study period, there were 5,563 study visits: 4,169 in the antenatal period and 1,394 at delivery. A total of 4,703 sample collections were made. Using our predefined sample selection criteria and removing duplicates, we selected 2,495 samples for analysis (Figure 2). All selected samples were analyzed in the laboratory and the results reported. These 2,495 samples were from 1,811 unique participants. The study participants were generally young with the majority in the 19 to 24 years group, reflecting the young age of child-bearing women in the study setting. Majority of the women had normal BMI at enrolment but 27.22% were overweight. The prevalence of smoking or any tobacco use was very low at less than 0.36%. Other participant characteristics are outlined in Table 1.

**Table 1:** Characteristics of pregnant women selected for the seroprevalence analysis*.

Characteristic*		(*N* = 1811**)	
		*n*	%
Maternal age (years), age groups	<19	56	3.09
	19–24	633	34.95
	25–29	517	28.55
	30–34	376	20.76
	35–39	182	10.05
	≥40	47	2.60
Maternal BMI categories (kg/m^2^)	Underweight, <18.5	64	3.53
	Normal, 18.5–24.9	923	50.97
	Overweight, 25.0–29.9	493	27.22
	Obese, ≥30.0	311	17.17
	Missing	20	1.10
Education Level	None	186	10.27
	Primary	979	54.06
	Secondary	450	24.85
	Higher	189	10.44
	Missing	7	0.39
Parity	0	494	27.28
	1 to 4	1,130	62.40
	≥ 5	158	8.72
	Missing	29	1.60
Marital status	Single	115	6.35
	Married/cohabiting	1,654	91.33
	Separated/divorced	32	1.77
	Widowed	3	0.17
	Missing	7	0.39
Health facility	Mariakani (urban)	973	53.73
	Rabai (rural)	838	46.27
	Missing	1	
Location of village of residence relative to the Mombasa-Nairobi highway	Not along the highway	1,184	65.38
	Along the highway	625	34.51
	Missing	2	0.11

*Characteristics assessed at first study visit in pregnancy.**N is the number of unique participants and not number of samples. Some participants had more than one sample selected during the study period.

Of all samples analyzed, 1,358 tested positive by Wantai [overall seropositivity of 54.4% (95% CI 52.45–56.39)], of which 811 [59.7%, (95% CI 57.06–62.34)] tested positive by the Euroimmun anti-SARS-CoV-2 NCP ELISA and 1,031 [75.9%, (95% CI 73.55–78.17)] tested positive by the Euroimmun anti-SARS-CoV-2 QuantiVac ELISA tests respectively; 964 samples [71.0%, (95% CI 68.49–73.39)] were positive by either of the 2 Euroimmun tests.

**Figure 3:**
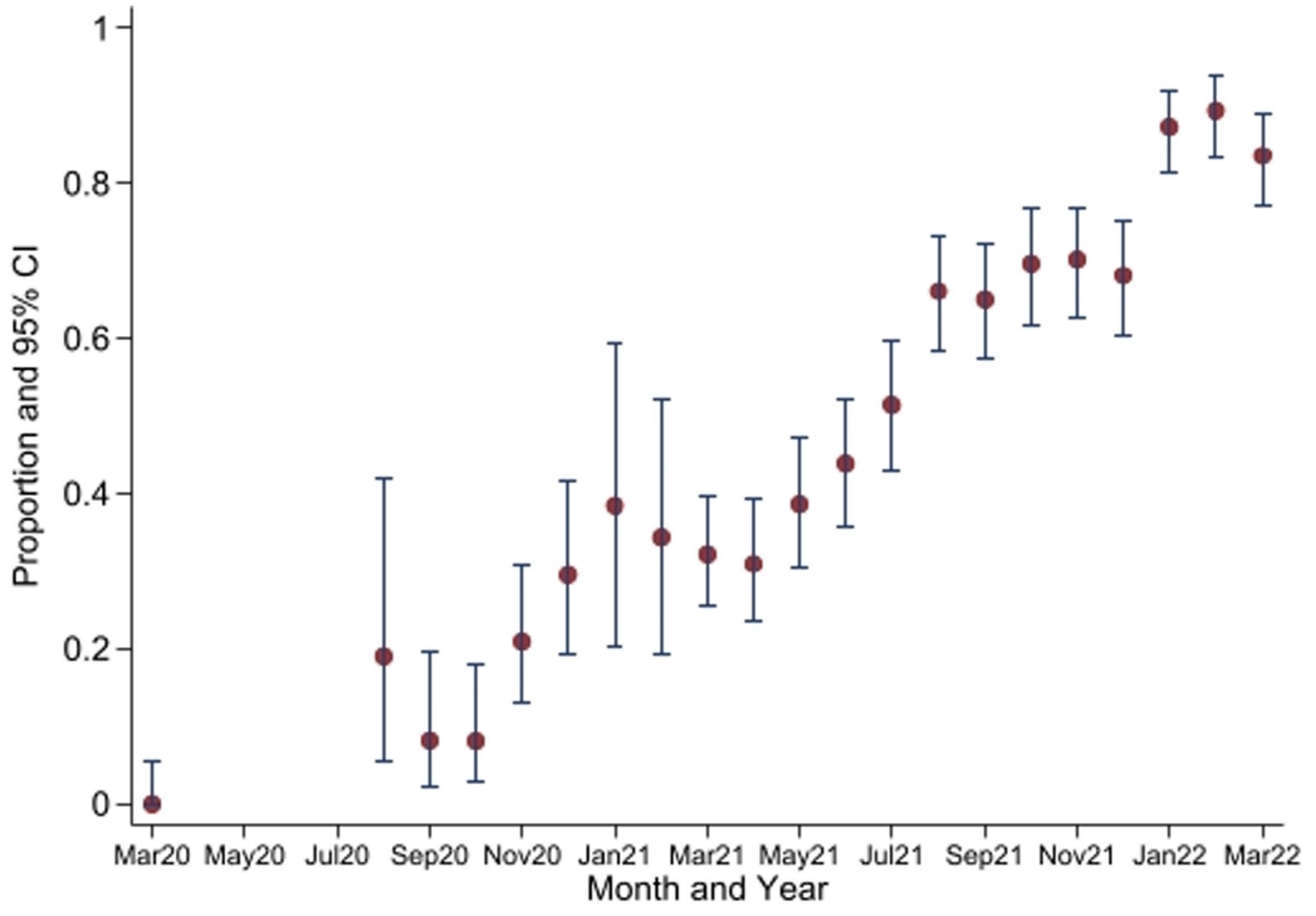
Seroprevalence of anti-SARS-CoV-2 antibodies by Wantai test between March 2020 and March 2022*. *No samples collected in April 2020 to July 2020. CI, Confidence Intervals.

**Figure 4:**
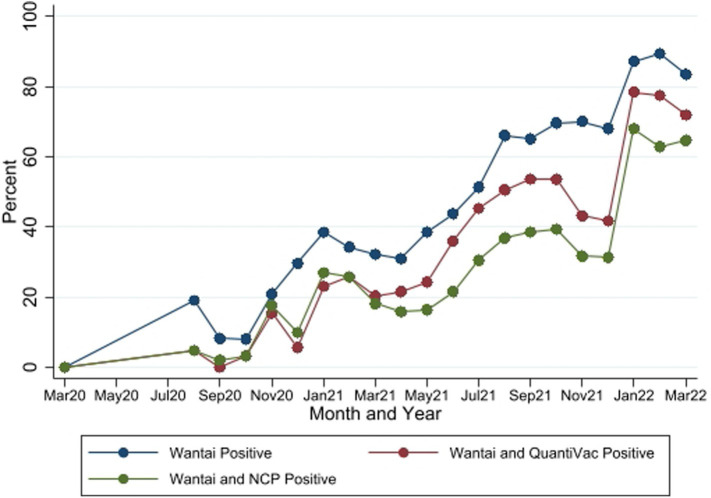
Seropositivity by the three SARS-CoV-2 antibody tests between March 2020 and March 2022. Wantai–SARS-CoV-2 ELISA kit (Wantai). NCP–Euroimmun anti-SARS-CoV-2 NCP ELISA test. QuantiVac–Euroimmun anti-SARS-CoV-2 QuantiVac ELISA test. Samples were tested with NCP and QuantiVac tests only if Wantai test was positive.

The seropositivity over time was highly variable with an overall trend of increasing seropositivity from a low of 0% [95%CI 0.00–0.06] in March 2020 to a high of 89.4% [95%CI 83.36–93.82] in Feb 2022 by Wantai (Figures 3, 4). The proportion of Wantai positive samples that tested positive for NCP ELISA and QuantiVac ELISA also increased over time. At the highest prevalence (Feb 2022), over 60% of the Wantai positive samples were positive by the Euroimmun anti-SARS-CoV-2 NCP ELISA.

There were no differences in seropositivity between the urban site and the rural site. Participants who resided in villages adjacent to the major highway (*vs* those away from the highway) had higher overall seroprevalence (Table 2).

**Table 2:** Comparison of seroprevalence of SARS-CoV-2 antibodies by various geographical characteristics.

Characteristic		Seropositive*, %, [95% confidence interval]	Adjusted Odds Ratio** [95% confidence interval]	*p* value***
Study facility	Rural	52.7 [49.78–55.57]	1.12 [0.93–1.34]	0.241
	Urban	56.0 [53.30–58.73]		
Location of village of residence relative to the Mombasa-Nairobi highway	Is not along the highway	50.4 [47.94–52.85]	1.48 [1.22–1.80]	<0.001
	Is along the highway	62.2 [58.87–65.46]		

*Wantai test. **adjusted for each month in the study period. ***χ^2^ test.

### 3.1 Comparisons between the monthly SARS-CoV-2 seroprevalence, COVID-19 cases in Kenya and important public health events

**Figure 5:**
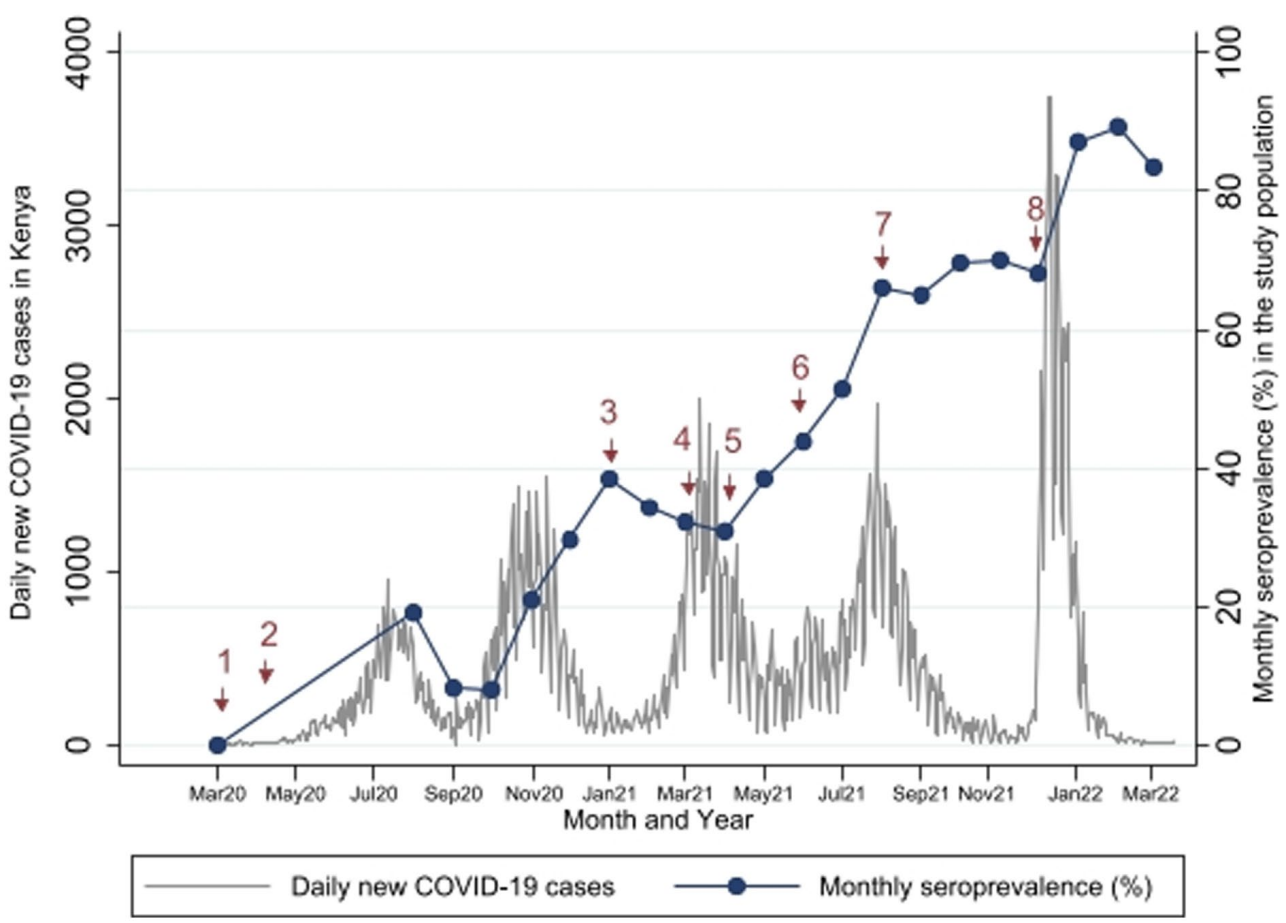
Study Seroprevalence* superimposed on the number of new COVID-19 cases per day in Kenya**. *Proportion of SARS-CoV-2 antibody positivity by Wantai test in each month. **Data on number of cases obtained from https://coronavirus.jhu.edu/map.html (17). Events at time points 1 to 8 are described in Table 3.

Comparisons between the monthly seroprevalence and the reported COVID-19 cases in Kenya are demonstrated on Figure 5. There were 5 COVID-19 waves in Kenya in the study period with the largest in December 2021. The graphs demonstrate a steep increase in seroprevalence after each wave. There was high transmission during the 5th wave in December 2021, which included confirmation of Omicron cases in Kenya (24), preceding a steep increase in seroprevalence from 68.1% [95% CI, 60.35–75.17] to 89.4% [95% CI, 83.36–93.82] in February 2022.

**Table 3:** Description of important public health events and measures.

Time point	Month	Event or public health measure(s)
1	March 2020	First COVID-19 case reported in Kenya. Restrictions to international travel. Closure of school and institutions of higher learning. First COVID-19 case reported in Kilifi county (19).
2	April 2020	6 April 2020–Cessation of movement in and out of the capital city (Nairobi metropolitan area) and coastal counties including Kilifi county (20).
3	January 2021	Reopening of schools (21).
4	March 2021	Start of vaccination in Kenya (Pregnant women not eligible). Start of vaccination in Kilifi county (22).
5	April 2021	Start of vaccination in the study area–Kaloleni and Rabai sub-counties.
6	June 2021	Start of vaccination campaigns in Kilifi County.
7	August 2021	20 August 2021. Recommendation by Ministry of Health to vaccinate all pregnant women (23).
8	December 2021	15 Dec 2021 - First Omicron case reported in Kenya (24).

*Numbers 1 to 8 correspond to public health events at specific time points shown on Figure 5.

Comparisons with important public health measures relevant to SARS-CoV-2 control in the general population and in pregnant women are displayed in Figure 5 with accompanying descriptions of the time points in Table 3. Vaccination started in the study area in April 2021 (time point 5), but these initial vaccinations were for health workers and other high risk populations - pregnant women were largely excluded. In August 2021 (time point 7), the Ministry of Health made a recommendation to vaccinate all pregnant women (23) but there is only a slight increase in seroprevalence in the months that followed (Sep to Dec 2021). The seroprevalence rose again sharply in January 2022 after the omicron wave.

The cumulative reported PCR positive cases in Kilifi County by March 2022 was 7,100 (18) against an estimated county population of 1.45 million (24) – 0.49% positivity.

## 4 Discussion

### 4.1 Summary of findings

The overall seroprevalence of SARS-CoV-2 antibodies was 54.4% based on the Wantai kit that detects both IgG and IgM antibodies to the spike protein. The monthly seroprevalence showed a stepwise increase over time that correlated with the COVID-19 waves in Kenya, suggesting that the increase in seroprevalence was driven by transmission of the infection. In this setting, the pandemic evolved rapidly and over 89% of the population was exposed within 23 months from the introduction of the virus. The highest seroprevalence (89.7%) was recorded in February 2022, a likely consequence of the Omicron variant which drove the fifth wave in Kenya toward the end of 2021 (24). This seroprevalence is over 180 times higher than the cumulative reported PCR positivity for Kilifi County (25). Considering the low SARS-CoV-2 vaccination coverage in Kenya during the study period (25), the reported prevalence probably indicates infection induced SARS-CoV-2 antibodies. This is supported by the steep increase in seroprevalence after each wave. At the highest prevalence, over 60% of the Wantai positive samples were confirmed to have antibodies against the nucleocapsid that occur only due to natural infection (26).

This study reports seroprevalence in pregnant women but the findings can be used to draw inferences on the general population in the study area. Pregnant women interact regularly with the rest of the population and are more similar to the population in their socioeconomic activities and status. This is in contrast to other special groups such as blood donors, health care workers and truck drivers who have been sampled in previous seroprevalence studies (27–30). In Kilifi, there is high antenatal clinic attendance (16) and a high fertility rate (31) meaning a large proportion of the population will be pregnant at any one time. The participants enrolled to the cohort were recruited from health facilities, the majority having presented for routine antenatal and delivery care.

Studies reporting on temporal trends of SARS-CoV-2 seroprevalence have shown varying prevalence at various epochs during the pandemic and different ‘rates of change’. A study conducted among pregnant women attending antenatal care in 3 health facilities in rural Eastern Ethiopia (April 2020 to March 2021) reported a seroprevalence of 5.7% with a slower rate of rise in seroprevalence over time (7). The highest seroprevalence was 11.8% in February 2021 compared with a seroprevalence of 35% [95% CI 19.1–52.2] in our study at the same time point. A similar study conducted among pregnant women attending antenatal care in 3 referral hospitals in Kenya, including the Kilifi County Hospital, between August 2020 and October 2021showed increasing seroprevalence with the prevalence for Kilifi County Hospital being comparable with our study findings, while the national referral hospital serving a predominantly urban Nairobi population had higher seroprevalence (6). Our study population is more rural than that of the Kilifi County Hospital catchment.

Our findings show a higher seroprevalence in the villages adjacent to the major highway in the study area. Cross-country travel that occurs along the highway could have driven the higher infection rates particularly in the earlier months of the pandemic. Studies of truck drivers have shown seropositivity rates of as high as 39.6% in October 2020 (28), compared with a seropositivity rate of 8.0% at a similar time point in our study. This is similar to the spread of Ebola and HIV along roads (32, 33) and should be considered in the design of specific mitigation measures for future health system shocks.

Of the 1,358 samples that tested positive with the Wantai kit, only 59.7% were positive for Euroimmun anti-SARS-COV-2 nucleocapsid antibodies which are produced following natural infection and not vaccination (26). However, alternative explanations may be the higher sensitivity of the Wantai (96.7%) compared with the Euroimmun IgG kit (78%) (34) and the fact that antibodies against the nucleocapsid protein wane more quickly than those against the spike protein (35). Use of test kits that target antibodies against the spike protein are preferred for population based seroprevalence studies especially where SARS-CoV-2 vaccine uptake is low (36). Vaccination rates in Kilifi county were low with only 14% of the entire population vaccinated as of 31st March 2022 (37) and likely lower vaccination rates among pregnant women.

### 4.2 Strengths and limitations of the study

#### 4.2.1 Strengths

This study has several strengths. The data and samples were prospectively collected and processed using standardized approaches both before and during the pandemic. To determine seroprevalence, we utilized the World Health Organization-recommended and high-specificity Wantai test kit (38). Standardized sample analysis was conducted using automated ELISA and with adequate quality control. Our sampling ensured good representation of the primary cohort with over 40% of available samples processed and a balanced representation across the various study months.

#### 4.2.2 Study limitations

Even though the samples selected for this study are representative of the primary cohort study, the cohort study is not fully representative of the population in Kilifi County as the recruitment was health facility based. The majority of the women who attend antenatal care in these facilities reside in areas closer to the health facilities with a smaller proportion of women being referred from further away. Even in the rural facility, the areas adjacent to the facility are more developed and have a denser population. The temporary cessation of study activities due to the pandemic resulted in fewer samples and wide confidence intervals in the affected months. We mitigated this risk by oversampling in the months with few samples (100% sampling fraction from months with <100 samples). Seroprevalence studies in pregnant women can be used to draw inferences on the general population but caution should still be exercised as the immune response and antibody production in pregnant women is different (39).

We did not collect individual data on vaccination status limiting inferences that can be drawn on the possible impact of vaccination on the seroprevalence. A detailed vaccination history would also have enabled a better estimation of the sensitivity and specificity of the nucleocapsid test. However, the high proportion of samples with nucleocapsid antibodies likely confirms that the SARS-CoV-2 antibodies were predominantly a result of infection and not vaccination.

### 4.3 Clinical implications for pregnant women and maternity services

Our study neither reports on clinical outcomes of the pregnancies, nor does it try to draw associations between the seroprevalence and individual participant characteristics. Our goal for this study was to demonstrate the seroprevalence at each point in time and from largely asymptomatic participants.

The high seroprevalence rates reported particularly in the latter months of the study period reveal high transmission of SARS-CoV-2. SARS-CoV-2 infection is known to increase adverse outcomes of pregnancy (40–42). Analysis of pregnancy outcome data would help shed light on SARS-CoV-2 related maternal and perinatal morbidity and mortality and how this evolved across the different waves. Vaccination of pregnant women in Kenya was only encouraged after August 2021 (23), and even then, uptake remained low (43). Vaccination of pregnant women in this setting could have prevented the high infection rates and likely reduced adverse pregnancy related complications which are known to occur more frequently in unvaccinated women (44).

This study highlights the significant difference in the reported COVID-19 cases (PCR confirmed) and the proportion of antibody positive persons detected in this study. Health systems in areas with limited access to laboratory services should invest more on seroprevalence studies as these can yield more informative data to inform any public health control measures. Seroprevalence studies would also be useful in settings where an infectious disease largely asymptomatic, as it was for SARS-CoV-2 in many parts of Africa (3).

We recommend continued sero-surveillance of SARS-CoV-2 especially with the declining PCR testing and we suggest sampling of pregnant women as an alternative and low-cost approach to estimate population level seroprevalence.

### 4.4 Implications for research

This study adds to the body of data on seroprevalence of SARS-CoV-2 in a cohort of pregnant women and reports the seroprevalence over a long duration of time (2-year period), giving insight into the evolution of the pandemic in this population and enabling inferences to be made on the general population in the same geographical setting. This study demonstrates the utilization of stored biological samples collected prospectively as part of a pregnancy cohort study to demonstrate seroprevalence from before the onset of a new infection (SARS-CoV-2) and through the evolution of the pandemic, demonstrating the value addition that biobanking can bring to clinical research.

## 5 Conclusion

The steep rise in seroprevalence of SARS-CoV-2 reported in this paper is evidence of a rapidly evolving infection with high transmission rates in this setting in Kenya. A majority of the population were exposed to SARS-CoV-2 within 23 months of the introduction of the virus.

## Data availability statement

The raw data supporting the conclusions of this article will be made available by the authors, without undue reservation.

## Ethics statement

The PRECISE study received ethical approval from the Aga Khan University Institutional Scientific and Ethics Review Committee (2018/REC-74) and King’s College London BDM Research Ethics Subcommittee (Ref HR-17/18–7855). The studies were conducted in accordance with the local legislation and institutional requirements. All participants gave written informed consent for study participation, biological sample collection and storage and use of the data and samples for future research.

## Group members of the PRECISE Network

Members of the PRECISE Network include: Patricia Okiro, Consolata Juma, Marvin Ochieng, Emily Mwadime, Esperança Sevene, Corssino Tchavana, Salesio Macuacua, Anifa Vala, Helena Boene, Lazaro Quimice, Sonia Maculuve, Eusebio Macete, Inacio Mandomando, Carla Carillho, Umberto D’Alessandro, Anna Roca, Hawanatu Jah, Andrew Prentice, Melisa Martinez-Alvarez, Brahima Diallo, Abdul Sesay, Sambou Suso, Baboucarr Njie, Fatima Touray, Yahaya Idris, Fatoumata Kongira, Modou F.S. Ndure, Gibril Gabbidon, Lawrence Gibba, Abdoulie Bah and Yorro Bah, Laura A. Magee, Hiten Mistry, Marie-Laure Volvert, Thomas Mendy, Lucilla Poston, Jane Sandall, Rachel Tribe, Sophie Moore, Tatiana T. Salisbury, Donna Russell, Prestige T. Makanga, Liberty Makacha, Reason Mlambo, Aris Papageorghiou, Alison Noble, Hannah Blencowe, Veronique Filippi, Joy Lawn, Matt Silver, Joseph Waiswa, Ursula Gazeley, Judith Cartwright, Guy Whitley, Sanjeev Krishna, Marianne Vidler, Jing (Larry) Li, Jeff Bone, Mai-Lei (Maggie) W Kinshella, Domena Tu, Ash Sandhu, Kelly Pickerill, and Ben Barratt.

## Group members of The periCOVID-Africa group

Members of the periCOVID-Africa group include Bridget Freyne, Kondwani Kawaza, Samantha Lissauer, Halvor Sommerfelt, Melanie Etti, Philippa Musoke, Robert Mboizi, Stephen Cose, Victoria Nankabirwa, Lauren Hookham, Joseph Ouma, Gordon Rukondo, Madeleine Cochet, Merryn Voysey, and Liberty Cantrell.

## Author contributions

AK: Conceptualization, Data curation, Formal analysis, Funding acquisition, Methodology, Project administration, Supervision, Visualization, Writing – original draft, Writing – review & editing. GO: Conceptualization, Data curation, Formal analysis, Investigation, Methodology, Supervision, Visualization, Writing – original draft, Writing – review & editing. AM: Data curation, Formal Analysis, Investigation, Methodology, Writing – original draft, Writing – review & editing. JM: Data curation, Investigation, Methodology, Writing – original draft, Writing – review & editing. IM: Data curation, Formal Analysis, Investigation, Methodology, Writing – original draft, Writing – review & editing. MM: Data curation, Formal analysis, Writing – original draft, Writing – review & editing. OW: Data curation, Methodology, Writing – review & editing. GM: Methodology, Project administration, Supervision, Writing – review & editing. GK: Conceptualization, Data curation, Methodology, Writing – original draft, Writing – review & editing. RC: Funding acquisition, Project administration, Writing – review & editing. PvD: Conceptualization, Funding acquisition, Methodology, Project administration, Writing – review & editing. KD: Conceptualization, Funding acquisition, Methodology, Project administration, Writing – review & editing. MT: Conceptualization, Funding acquisition, Methodology, Project administration, Writing – review & editing.
