# Corrigendum: SARS-CoV-2 seroprevalence in pregnant women in Kilifi, Kenya from March 2020 to March 2022

**DOI:** 10.3389/fpubh.2024.1500467

**Published:** 2024-10-14

**Authors:** Angela Koech, Geoffrey Omuse, Alex G. Mugo, Isaac G. Mwaniki, Joseph M. Mutunga, Moses W. Mukhanya, Onesmus Wanje, Grace M. Mwashigadi, Geoffrey G. Katana, Rachel Craik, Peter von Dadelszen, Kirsty Le Doare, Marleen Temmerman, periCOVID-Africa, The PRECISE Network

**Affiliations:** ^1^Centre of Excellence in Women and Child Health, Aga Khan University, Nairobi, Kenya; ^2^Department of Obstetrics and Gynaecology, Aga Khan University, Nairobi, Kenya; ^3^Department of Pathology, Aga Khan University, Nairobi, Kenya; ^4^Kilifi County Department of Health and Sanitation Services, Kilifi, Kenya; ^5^Department of Women and Children's Health, Kings College London, London, United Kingdom; ^6^Faculty of Medicine and Health Sciences, Ghent University, Ghent, Belgium; ^7^St. George's University of London, London, United Kingdom

**Keywords:** SARS-CoV-2, COVID-19, seroprevalence, pregnancy, Kenya, antibodies

In the published article, there was an error in Figure 4 as published. The reported antibody seropositivity rates by Wantai and QuantiVac (the maroon line) are incorrect. This error arose from incorrect configuration of cut-off values for determination of qualitative results for the test. The cutoff values configured into the analyzer for the EuroImmun QuantiVac test were for BAU/ml but the results were reported in RU/ml. The corrected Figure 4 and its caption appear below.

**Figure 4:**
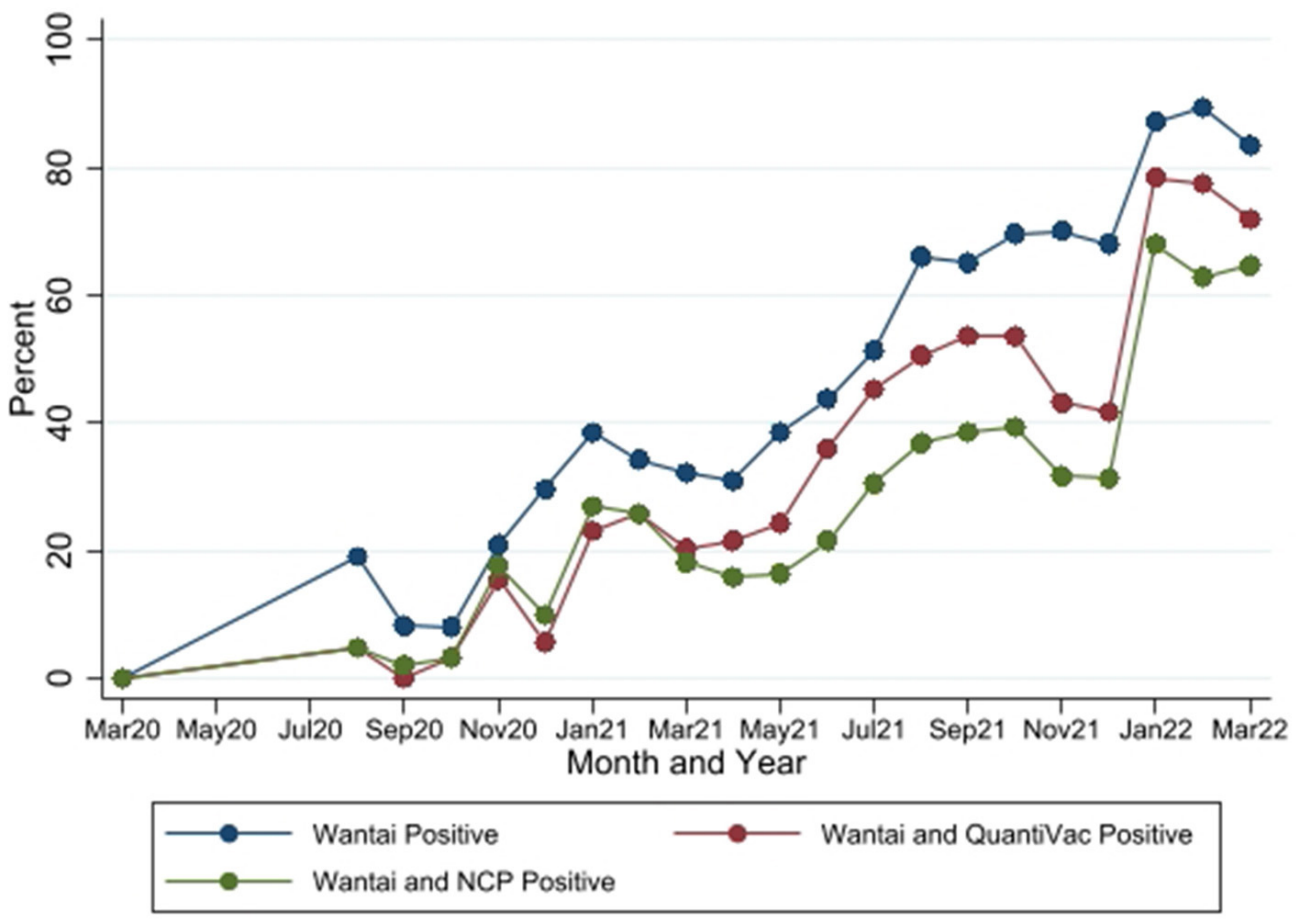
Seropositivity by the three SARS-CoV-2 antibody tests between March 2020 and March 2022. Wantai–SARS-CoV-2 ELISA kit (Wantai). NCP–Euroimmun anti-SARS-CoV-2 NCP ELISA test. QuantiVac–Euroimmun anti-SARS-CoV-2 QuantiVac ELISA test. Samples were tested with NCP and QuantiVac tests only if Wantai test was positive.

In the published article, there was an error. The reported antibody seropositivity for the EuroImmun QuantiVac test was incorrect. This error arose from incorrect configuration of cut-off values for determination of qualitative results for the test. The cutoff values configured into the analyzer for the EuroImmun QuantiVac test were for BAU/ml but the results were reported in RU/ml.

A correction has been made to **Abstract**, *Results*, Paragraph 3. This sentence previously stated:

“Of the Wantai test-positive samples, 59.7% [95% CI 57.06–62.34] tested positive by the Euroimmun anti-SARS-CoV-2 NCP test and 37.4% [95% CI 34.83–40.04] tested positive by the Euroimmun anti-SARS-CoV-2 QuantiVac test.”

The corrected sentence appears below:

“Of the Wantai test-positive samples, 59.7% (95% CI 57.06–62.34) tested positive by the Euroimmun anti-SARS-CoV-2 NCP test and 75.9% (95% CI 73.55–78.17) tested positive by the Euroimmun anti-SARS-CoV-2 QuantiVac test.”

In the published article, there was an error. The reported antibody seropositivity for the EuroImmun QuantiVac test was incorrect. This was reported as 37.4% (95% CI 34.8–40.04%). The new corrected result is 75.9% (95% CI 73.55–78.17%).

A correction has been made to **Results**, Paragraph 2. This sentence previously stated:

“Of all samples analyzed, 1,358 tested positive by Wantai [overall seropositivity of 54.4% (95% CI 52.45–56.39)], of which 811 [59.7%, (95% CI 57.06–62.34)] tested positive by the Euroimmun anti-SARS-CoV-2 NCP ELISA and 508 [37.4%, (95% CI 34.83–40.04)] tested positive by the Euroimmun anti-SARS-CoV-2 QuantiVac ELISA tests respectively; 964 samples [71.0%, (95% CI 68.49–73.39)] were positive by either of the 2 Euroimmun tests.”

The corrected sentence appears below:

“Of all samples analyzed, 1,358 tested positive by Wantai [overall seropositivity of 54.4% (95% CI 52.45–56.39)], of which 811 [59.7%, (95% CI 57.06–62.34)] tested positive by the Euroimmun anti-SARS-CoV-2 NCP ELISA and 1,031 [75.9%, (95% CI 73.55–78.17)] tested positive by the Euroimmun anti-SARS-CoV-2 QuantiVac ELISA tests respectively; 964 samples [71.0%, (95% CI 68.49–73.39)] were positive by either of the 2 Euroimmun tests.”

The authors apologize for these errors and state that this does not change the scientific conclusions of the article in any way. The original article has been updated.

